# Clinical validation of a new thermodilution system for the assessment of cardiac output and volumetric parameters

**DOI:** 10.1186/cc11366

**Published:** 2012-05-30

**Authors:** Nicholas Kiefer, Christoph K Hofer, Gernot Marx, Martin Geisen, Raphaël Giraud, Nils Siegenthaler, Andreas Hoeft, Karim Bendjelid, Steffen Rex

**Affiliations:** 1 Department of Anesthesiology and Intensive Care Medicine, University of Bonn, Sigmund-Freud-Straße 25, 53105 Bonn, Germany; 2 Institute of Anaesthesiology and Intensive Care Medicine, Triemli City Hospital Zurich, Birmensdorferstrasse 497, 8063 Zürich, Switzerland; 3 Department of Anaesthesiology, University Hospital Aachen, RWTH Aachen Pauwelsstraße 30, 52074 Aachen, Germany; 4 Intensive Care Service, Geneva University Hospitals, Rue Gabrielle-Perret-Gentil 4, 1211 Genève 14, Switzerland

**Keywords:** cardiac output, concordance, extravascular lung water, global end diastolic volume, monitoring, transpulmonary thermodilution

## Abstract

**Introduction:**

Transpulmonary thermodilution is used to measure cardiac output (CO), global end-diastolic volume (GEDV) and extravascular lung water (EVLW). A system has been introduced (VolumeView/EV1000™ system, Edwards Lifesciences, Irvine CA, USA) that employs a novel algorithm for the mathematical analysis of the thermodilution curve. Our aim was to evaluate the agreement of this method with the established PiCCO™ method (Pulsion Medical Systems SE, Munich, Germany, clinicaltrials.gov identifier: NCT01405040)

**Methods:**

Seventy-two critically ill patients with clinical indication for advanced hemodynamic monitoring were included in this prospective, multicenter, observational study. During a 72-hour observation period, 443 sets of thermodilution measurements were performed with the new system. These measurements were electronically recorded, converted into an analog resistance signal and then re-analyzed by a PiCCO_2_™ device (Pulsion Medical Systems SE).

**Results:**

For CO, GEDV, and EVLW, the systems showed a high correlation (r^2 ^= 0.981, 0.926 and 0.971, respectively), minimal bias (0.2 L/minute, 29.4 ml and 36.8 ml), and a low percentage error (9.7%, 11.5% and 12.2%). Changes in CO, GEDV and EVLW were tracked with a high concordance between the two systems, with a traditional concordance for CO, GEDV, and EVLW of 98.5%, 95.1%, and 97.7% and a polar plot concordance of 100%, 99.8% and 99.8% for CO, GEDV, and EVLW, respectively. Radial limits of agreement for CO, GEDV and EVLW were 0.31 ml/minute, 81 ml and 40 ml, respectively. The precision of GEDV measurements was significantly better using the VolumeView™ algorithm compared to the PiCCO™ algorithm (0.033 (0.03) versus 0.040 (0.03; median (interquartile range), *P *= 0.000049).

**Conclusions:**

For CO, GEDV, and EVLW, the agreement of both the individual measurements as well as measurements of change showed the interchangeability of the two methods. For the VolumeView method, the higher precision may indicate a more robust GEDV algorithm.

**Trial registration:**

clinicaltrials.gov NCT01405040.

## Introduction

Measurement of cardiac output (CO) and other parameters that guide cardiovascular therapy is paramount for the hemodynamic management of critically ill patients. Pulmonary thermodilution and pulmonary artery occlusion pressure have been the mainstay of advanced hemodynamic monitoring for decades. However, studies revealed conflicting results regarding the use of the pulmonary artery catheter (PAC) in order to improve the morbidity and mortality of critically ill patients [1-3] and as a result, PAC use is decreasing [4].

Meanwhile, less invasive techniques for CO measurement have emerged, such as transpulmonary thermodilution (TPTD), which has been validated in a variety of clinical settings (a review of all available validation studies is provided in [5]). In addition to CO measurements, TPTD provides volumetric hemodynamic parameters, that is, global end-diastolic volume (GEDV) and extravascular lung water (EVLW). GEDV has been shown to be a more reliable parameter of intravascular volume status when compared with the standard pressure preload parameters [6-8]. EVLW is a sensitive marker of pulmonary edema and a valuable prognostic parameter [9,10]. TPTD can be reliably performed at bedside [11] and has been successfully implemented in algorithms for goal directed hemodynamic therapy [12].

Recently, a new TPTD system has been developed and introduced into clinical practice. It consists of a specific thermistor-tipped arterial catheter, the VolumeView™ catheter and EV 1000™ monitoring platform (Edwards Lifesciences, Irvine, CA, USA) and employs a novel algorithm for the mathematical analysis of the thermodilution curve (details are described in the methods section). So far, the system has been validated in an animal model only [13]. That study revealed good agreement with measurements performed by the PiCCO™ system (Pulsion Medical Systems SE, Munich, Germany) that has been the only commercially available device based on TPTD.

The aim of the present multi-center clinical study was to assess the agreement of the new EV 1000™ system with the PiCCO™ system in a clinical setting of a mixed ICU population.

## Materials and methods

This prospective observational study was conducted in four hospital centers in Germany and Switzerland (Aachen, Bonn, Geneva, and Zurich). Approval was obtained from local institutional review boards at all participating institutions (Ethik-Kommission an der Medizinischen Fakultät der Rheinisch-Westfälischen Technischen Hochschule Aachen, Aachen, Germany; Ethikkommission an der Medizinischen Fakultät der Rheinischen Friedrich-Wilhelms-Universität Bonn, Bonn, Germany; Commission Centrale d'Ethique de la recherche sur l'être humain, Hôpitaux Universitaires de Genève, Genève, Switzerland; Ethikkommission der beiden Stadtspitäler Triemli und Waid, Kantonale Ethikkommission des Kantons Zürich, Zürich, Switzerland). The trial is registered at a public registry (clinicaltrials.gov identifier: NCT01405040). All patients or their legal representatives gave written informed consent. We enrolled patients who had been admitted to an intensive care unit and required advanced hemodynamic monitoring by TPTD according to the treating clinician after the patients, or their legal representatives, gave written informed consent. Patients younger than 18 years old were not eligible for the study; neither were patients with a body weight less than 40 kg, according to the manufacturer's directions for use. Other exclusion criteria were significant aortic regurgitation in the patient's history, treatment with an intra-aortic balloon pump, participation in an investigational drug or device study interfering with the endpoints of this study and a known or potential pregnancy. During a 72-hour observation period, TPTD measurements were performed with the VolumeView™/EV1000™ system as clinically indicated and data were electronically recorded and then reanalyzed offline by a PiCCO_2_™ System (Pulsion Medical Systems SE, Munich, Germany, software Version 8.0.0.6). All patients were treated at the discretion of the ICU staff in charge; there was no specific protocol for any intervention.

### Transpulmonary thermodilution measurements

A VolumeView™ catheter (Edwards Lifesciences) was inserted into the left or right femoral artery and connected to the EV1000™ monitoring system (Edwards Lifesciences, software version 1.0). Thermodilution measurements were performed in sets of at least three consecutive injections of 20 ml cold saline, randomly distributed over the respiratory cycle. As required by the EV1000 monitor's software, individual boluses of each set had to be manually validated by the attending physician before they were included in the set. By protocol, bolus sets that contained a bolus that differed by more than 15% from the mean within the set were considered faulty and excluded from the analysis. Also, boluses for which either system's internal artifact recognition algorithm reported an error were not included. All hemodynamic data were electronically recorded at 500 Hz internally on the EV1000™ system and downloaded for analysis. Blood temperature and injectate temperature data were converted into an analog output signal by LabView software (National Instruments, Austin, TX, USA) and a multichannel input/output module (NI 9263, National Instruments). That analog signal was then transformed into a resistance signal, which can be read as blood and injectate temperature by a PiCCO_2_™ System (Pulsion Medical). This set-up was validated for the relevant range of blood and injectate temperatures by comparison of input temperatures with the temperatures read and stored by the PiCCO_2_™ device.

### Algorithms for CO, GEDV and EVLW assessment

Both the PiCCO™ and the VolumeView™/EV1000™ system use the Stewart-Hamilton-equation [14] to calculate the thermodilution derived cardiac output (CO_TD_):

(1) $$CO_{TD}=\frac{V_{i}\cdot \left(T_{blood}-T_{injectate}\right)\cdot k}{\int \text{Δ}T_{blood}\cdot dt}$$

*V_i _*is the volume of the injectate, *T_blood _*and *T_injectate _*are blood and injectate temperatures, whereas *k *is a constant proportional to the specific heat and weight of the blood and the injectate, and *t *is the time from injection of the bolus.

**Figure 1:**
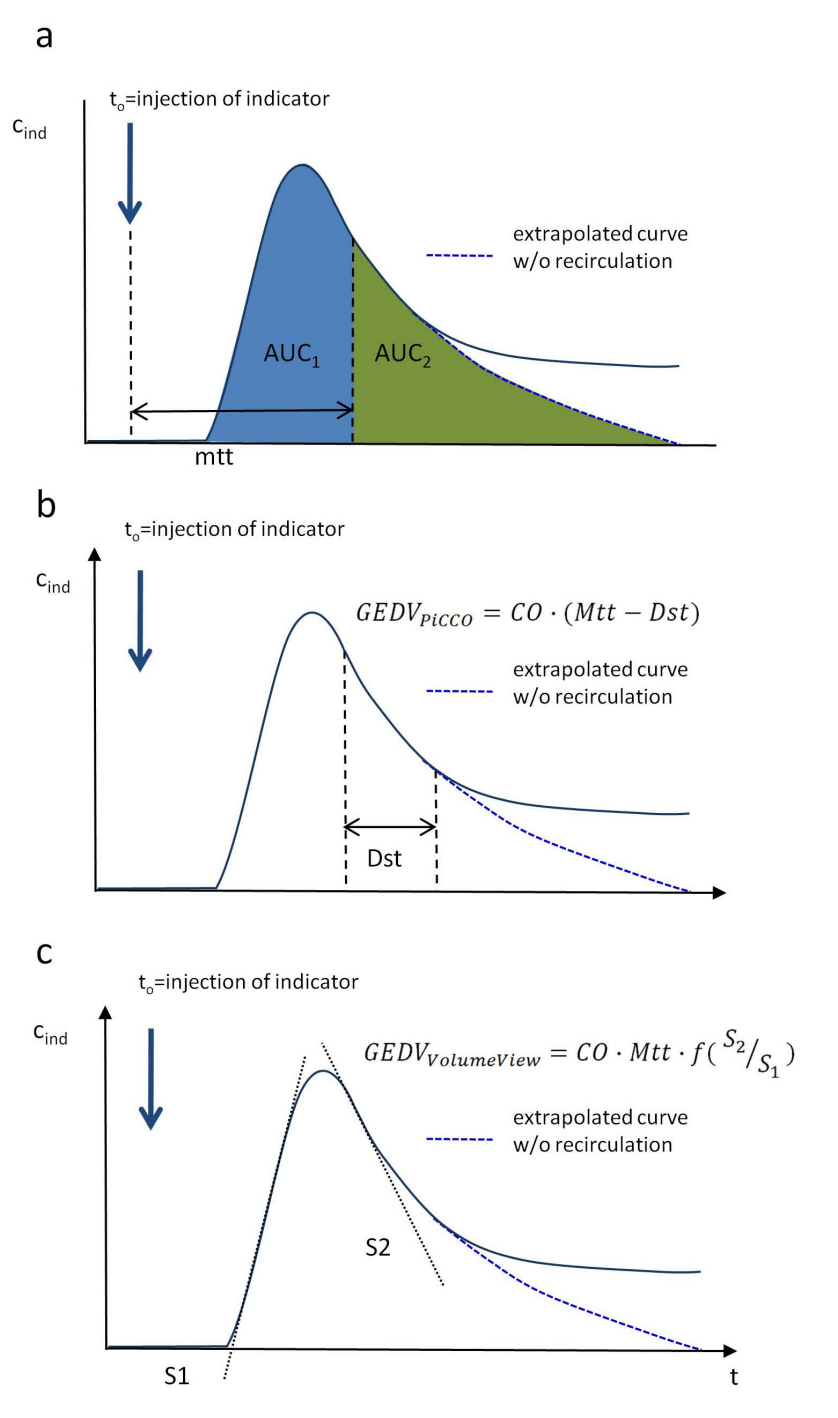
**Mathematical analysis of the thermodilution curve**. Panel **a**) Both algorithms rely on mean transit time (Mtt), the time required for half of the indicator to pass the thermistor in the femoral artery. Mtt divides the area under the curve (AUC) into two areas of the same size (AUC_1 _and AUC_2_). Panel **b**) Downslope time (Dst) is part of the PiCCO™ GEDV algorithm. It is the time of the temperature decay between two set points in the thermodilution curve, for example, 80% to 40%. Theoretically, the decay is mono-exponential, so it can be measured at any time point after the peak and be adjusted by a constant factor. Panel **c**) The VolumeView™ algorithm relies on maximum up-slope (S_1_) and maximum down-slope (S_2_) of the dilution curve. This approach may be less sensitive to early recirculation and thermal noise.

The systems use different algorithms to assess GEDV (Figure 1): The algorithm implemented in the PiCCO_2_™ System applies mean transit time (Mtt) and downslope time (Dst) according to the Newman paradigm [15]:

(2) $$GEDV_{PiCCO}=CO\cdot \left(Mtt-Dst\right)$$

The VolumeView™/EV1000™ system employs a newly developed algorithm requiring the determination of the maximum up-slope (*S_1_*) and down-slope (*S_2_*) of the thermodilution curve, and a proprietary function (*f*, Figure 1):

(3) $$GEDV_{VolumeView}=CO\cdot Mtt\cdot f\left(S_{1}\;/)\;S_{2}\right)$$

The algorithms for EVLW assessment are equivalent for both systems. However, they rely on GEDV that is calculated in different ways according to formulas 2 and 3:

(4) $$EVLW_{PiCCO}=CO\cdot Dst-\left(0.25\cdot GEDV_{PiCCO}\right)$$

(5) $$EVLW_{VolumeView}=CO\cdot Dst-\left(0.25\cdot GEDV_{VolumeView}\right)$$

Of note, formula (4) is equivalent to the typical EVLW algorithm (11) but has been mathematically transformed to facilitate comparison of the algorithms; details of that conversion are shown in Additional file 1.

### Statistical analysis

All thermodilution data were imported into an Excel™ spreadsheet (Microsoft, Redmond, WA, USA) that was used to calculate bias, limits of agreement, concordance and polar coordinates. All other statistical analyses were performed using PASW Statistics 18 (IBM, Armonk, NY, USA). Normal distribution of the data was tested using the Kolomogorov Smirnov test. A *P *< 0.05 was considered statistically significant. Normally distributed data are presented as mean ± standard deviation (SD), otherwise as median (interquartile range). The interquartile range is calculated as the difference between the third and the first quartile. Bias and limits of agreement were assessed as proposed by Bland and Altman [16] with the difference between the parameters measured by the two systems plotted against their mean. Bias refers to the mean difference and limits of agreement to the 95% confidence interval of the difference with correction for repeated measurements [17]. The percentage error is the relation of two standard deviations of the difference to the mean of both devices. To assume interchangeability, we adopted the 30% threshold proposed by Critchley and Critchley [18]. Paired measurements, which are two consecutive measurements using the same method, permit the quantification of the change within the measured parameter, for example, ΔCO. The difference expressed as a percentage of the mean is termed percentage change, concordance is the agreement of the direction of the change obtained from paired measurements by the two methods, expressed as the percentage of the total number of data points. Data with small changes are excluded from the analysis ('exclusion zone') because without significant change, data reflect only random effects and not actual change. We used an exclusion zone of 10%, which is smaller than recently suggested [19], in order to use a larger proportion of the data. Sufficient concordance to assume interchangeability was set to 95%, following the most conservative threshold proposed by Critchley *et al. *[19]. In addition, percentage changes were plotted in a polar diagram using the transformation described by Critchley *et al. *[20]. Briefly, percentage changes were converted into polar coordinates (angle and radius) that represent agreement as *θ*, the angle to line of identity. The line of identity, indicating equal changes obtained by the two systems, is rotated clockwise by 45 degrees in order to obtain a horizontal line:

(6) $$\theta =\left\{\begin{array}{cc} \left({\text{tan}}^{-1}\frac{{\text{Δ}}_{VolumeView}\left(\%\right)}{{\text{Δ}}_{PiCCO}\left(\%\right)}+k\right)\cdot 180^{\circ }\;/)\;\pi & \;if\Delta_{PiCCO}\neq 0\\ \left(\frac{\pi }{2}+k\right)\cdot 180^{\circ }\;/)\pi & \;\;if\Delta_{PiCCO}=0 \end{array}\right)$$

*k *is a constant used to correctly rotate radians by 45 degrees for data from different quadrants of the plot. When the percentage change of the test method is positive, the constant is -0.79, for a negative change 2.36. The case differentiation was introduced to avoid a division by zero. The distance of the polar coordinates from the centre represents the mean percentage change calculated by the two algorithms.

Concordance was calculated as the percentage of data within ± 10% 'tram line', a criterion chosen in analogy to the traditional concordance:

$$\left|\text{sin}\theta \cdot \frac{{\text{Δ}}_{PiCCO}\left(\%\right)+{\text{Δ}}_{VolumeView}\left(\%\right)}{2}\right|<10\%$$

Again, sufficient concordance to assume interchangeability was set to 95%, following the most conservative threshold proposed by Critchley *et al. *[18]. Radial limits of agreement (RLOA) are the 95% confidence interval of the *θ *of all data points outside a central exclusion zone. We chose a 10% exclusion zone in analogy to the traditional concordance. Mean *θ *was calculated after conversion of all angles within the left quadrants to the respective right quadrants, RLOA are mean *θ *± 1.96 SD. For interchangeability, the threshold was set to a mean angle smaller than ± 5° and RLOA smaller than ± 30, according to [18]. The coefficient of error (CE) of measurements using the GEDV and EVLW algorithms was determined for each set:

(8) $$CE_{GEDV}=\frac{SD_{GEDV}}{mean_{GEDV}\cdot \sqrt{n}}$$

*SD_GEDV _*is the SD within each set, *mean_GEDV _*is the mean and *n *the number of the repeated measurements. Precision refers to two times CE [21], thus a lower CE indicates higher precision. EVLW measurements were analyzed accordingly. The CE was not analyzed for CO, as both algorithms are identical.

## Results

Between March and December 2010, 72 patients were entered into the study. Their biometric and demographic data, as well as the reasons for admission to an ICU are presented in Table 1. During the observation period, the patients were mostly on assisted mechanical ventilation (62% of all scheduled visits) or breathing spontaneously (28%). On 9% of all scheduled visits they were on controlled mechanical ventilation. Median positive end-expiratory pressure was 6 cm H_2_O (5), median tidal volume of intubated patients was 500 (168) ml. Median of the patients' mean arterial pressure was 77 (15) mmHg. Vasopressors (noradrenaline, adrenaline, vasopressin) were administered during 76%, inotropes (dobutamine, adrenaline, milrinone, levosimendan) were administered during 33%, and nitroglycerine during 8% of all measurements. Four hundred and forty-three paired bolus sets with a median injectate temperature of 9 (3) °C were analyzed by both systems, allowing 370 paired change measurements. The median number of sets per patient was 10 (5). In one patient, only one single bolus set was recorded, precluding trend assessment. The observed range of CO, GEDV and EVLW values is displayed in Table 2.

**Table 1:** Demographic details and reason for ICU admission for all 72 patients.

	mean
age (years)	66 ± 12
gender (m/f)	51/21
height (cm)	172 ± 8
weight (kg)	82 ± 20
**reason for ICU Admission**	**Number**

post cardiac surgery	40
other (for example, respiratory failure, intracranial bleeding)	18
sepsis	7
post non cardiac surgery	4
ARDS	3

Data presented as absolute numbers or mean ± standard deviation. ARDS, acute respiratory distress syndrome; m/f, male/female.

**Table 2:** Hemodynamic parameters of all 72 patients.

	PiCCO™				VolumeView™			
	median	IQR	min	max	median	IQR	min	max
CO (ml/minute)	6.0	2.6	1.8	15.8	6.2	2.6	2.2	15.3
GEDV (ml)	1,,337	405	712	2,433	1,379	405	741	2,427
EVLW (ml)	566	257	265	2,132	589	246	313	2,015

CO, cardiac output; EVLW, extravascular lung water; GEDV. global end-diastolic volume; IQR, interquartile range; max, maximum value; min, minimum value.

**Figure 2:**
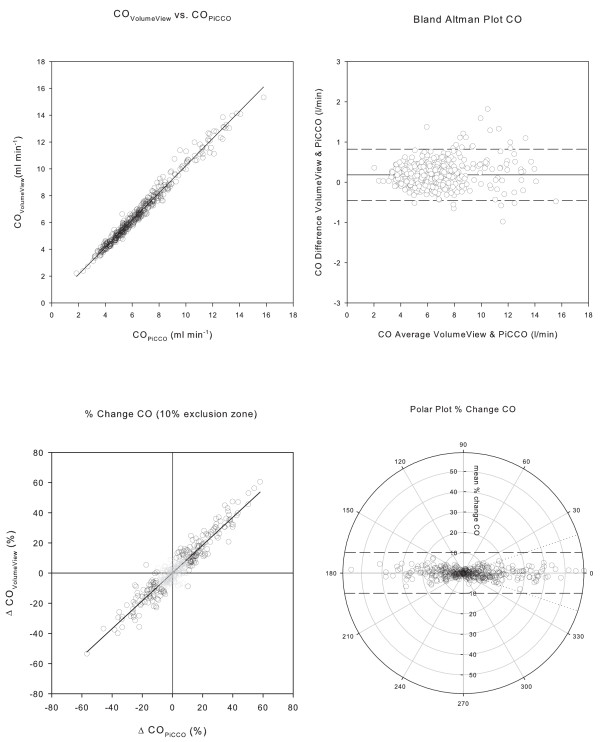
**Comparison of cardiac output measurements**. Panel **a**) Correlation between measurements of cardiac output (CO) with the VolumeView™/EV1000™ system and reanalysis with the PiCCO_2_™ system. Panel **b**) Bland Altman Plot, with the difference between the values derived from the two algorithms plotted against their mean. The solid line represents bias, the two dashed lines the upper and lower limit of agreement. Panel **c**) Concordance plot of percentage change. Data points within the 10% exclusion zone are grayed out. Panel **d**) Polar plot with distance from the center as mean change and *θ*, the angle with the horizontal axis, as agreement. The dashed tram line intersects the 90° axis at ± 10% and marks the limit of acceptable concordance. The dotted lines mark the radial limits of agreement.

**Figure 3:**
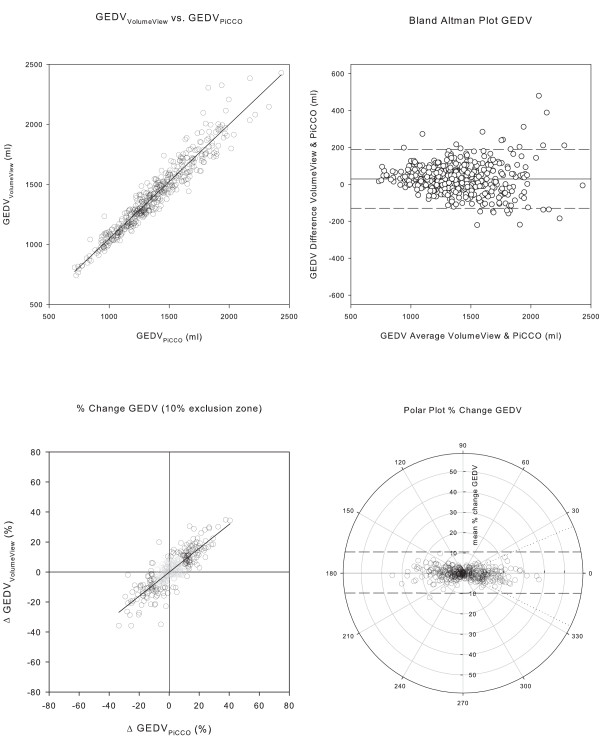
**Comparison of global end-diastolic volume measurements**. Panel **a**) Correlation between global end-diastolic volume (GEDV) computed with the PiCCO™ and the VolumeView™ algorithm. Panel **b**) Bland Altman Plot, with the difference between the values derived from the two algorithms plotted against their mean. The solid line represents bias, the two dashed lines the upper and lower limit of agreement. Panel **c**) Concordance plot of percentage change. Data points within the 10% exclusion zone are grayed out. Panel **d**) Polar plot with distance from the center as mean change and *θ*, the angle with the horizontal axis, as agreement. The dashed tram line intersects the 90° axis at ± 10% and marks the limit of acceptable concordance. The dotted lines mark the radial limits of agreement.

**Figure 4:**
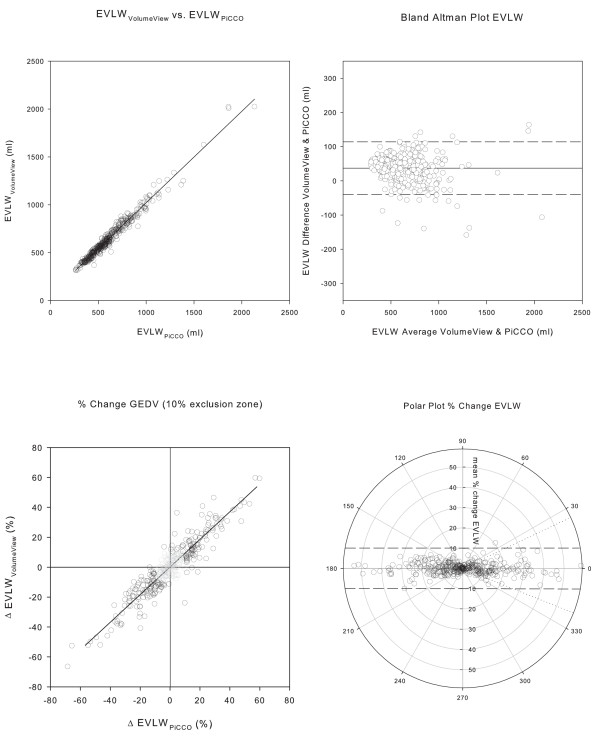
**Comparison of extravascular lung water measurements**. Panel **a**) Correlation between extravascular lung water computed with the PiCCO™ and the VolumeView™ algorithm. Panel **b**) Bland Altman Plot, with the difference between the values derived from the two algorithms plotted against their mean. The solid line represents bias, the two dashed lines upper and lower limit of agreement. Panel **c**) Concordance plot of percentage change. Data points within the 10% exclusion zone are grayed out. Panel **d**) Polar plot with distance from the center as mean change and *θ*, the angle with the horizontal axis, as agreement. The dashed tram line intersects the 90° axis at ± 10% and marks the limit of acceptable concordance. The dotted lines mark the radial limits of agreement.

The agreement of the two systems was high, exceeding all prerequisites to assume interchangeability for the measurement of CO, GEDV and EVLW. Regression analysis and Bland Altman plots of CO, GEDV and EVLW are depicted in Figures 2, 3, and 4a and 4b; corresponding data are presented in Table 3. The assessment of trending capability also demonstrated the interchangeability of the two systems. Four quadrant- and polar-plots are shown in Figures 2, 3 and 4c and 4d: the data are presented in Table 4. The precision of the novel VolumeView™/EV1000™ algorithm was significantly higher than that of the PiCCO algorithm for the calculation of GEDV (CE = 0.17 (0.02) versus 0.21 (0.02), *P *= 0.000039). Precision did not differ between the algorithms for the calculation of EVLW (0.22 (0.02) versus. 0.24 (0.02), *P *= 0.27 for the VolumeView™/EV1000™ and the PiCCO algorithms, respectively).

**Table 3:** Agreement between CO, GEDV and EVLW measurements by the VolumeView/EV1000™ and the PiCCO_2_™ system.

	r^2^	bias	LOA	% error
CO	0.981	0.2 ml/min	0.45 to 0.82 l/min	9.7%
GEDV	0.926	29.4 ml	-130 to 189 ml	11.5%
EVLW	0.971	36.8 ml	-40 to 113 ml	12.2%

CO, cardiac output; GEDV, global enddiastolic volume; EVLW, extravascular lung water; LOA, limits of agreement.

**Table 4:** Agreement between trends in CO, GEDV and EVLW measurements by the VolumeView/EV1000™ and the PiCCO_2_™ system.

	four quadrant plot		polar plots	
		
	conc_tot_	conc_10%_	concordance	radial LOA
CO	87.6%	98.5%	100%	0.31 ml/minute
GEDV	81.5%	95.1%	99.7%	81 ml
EVLW	88.6%	97.7%	99.7%	40 ml

CO, cardiac output; GEDV, global enddiastolic volume; EVLW, extravascular lung water; conc_tot_, concordance rate including all data points; conc_10%_, concordance rate using a 10% exclusion zone; LOA, limits of agreement.

## Discussion

This prospective, multi-center clinical study demonstrates that CO, GEDV and EVLW calculated by the newly introduced VolumeView/EV1000™ system are interchangeable with CO, GEDV and EVLW obtained using the PiCCO algorithms over a wide range of values and in various clinical situations, including low cardiac output syndrome, hyperdynamic state, hypo- and hyper-volemia, and severe pulmonary edema. Interchangeability was demonstrated both for the absolute measures with a percentage error well below 30% and for the detection of trends, with concordance in the four quadrant plot higher than 95%. Using the polar plot method, interchangeability was demonstrated both with polar plot concordance higher than 95% and RLOA smaller than ± 30°.

For calculation of CO, both methods employ the Stewart-Hamilton equation. Therefore, the close agreement of CO assessed by the two methods was not a surprise. It was substantially better than any premise postulated for interchangeability and superior to the agreement reported for other studies comparing CO monitoring systems [18]. Only minimal bias, very low percentage error and accurate trending capability were observed. Regression analysis showed good linearity over the entire data range. The residual disagreement can be interpreted as a result of minor methodological differences in the calculation of the area under the curve, which is part of the denominator in formula 1. These differences include, for example, how the thermodilution curve is truncated and how a recirculation-free curve is extrapolated.

For GEDV, different mathematical algorithms are used. The PiCCO_2_™ system employs a time constant derived from the down-slope of the thermodilution curve. The VolumeView™/EV1000 system, in contrast, uses both the up-slope and the down-slope of the thermodilution curve. Therefore, more information of the curve shape is taken into account so that, supposedly, the algorithm is less sensitive to recirculation and thermal noise. This assumption is supported by a significantly higher precision of that algorithm. For EVLW, interchangeability was also clearly demonstrated. Agreement was not as good as for CO measurements, although algorithms appear to be identical in both systems (formulas 4 and 5, Additional file 1). However, the algorithms use a differently calculated GEDV, which explains the slightly lower agreement. In contrast to GEDV measurements, higher precision of the VolumeView algorithm could not be demonstrated for EVLW. This may be due to the fact that identically calculated CO and Dst are part of the algorithms and outweigh differences in GEDV precision. The results of the present study show that the systems can be used interchangeably in various clinical situations and over a wide range of clinically relevant conditions, such as low or high cardiac output, hypo- and hyper-volemia and presence or absence of pulmonary edema. These results thus confirm the data from a recent animal validation [13], in which both systems, the PiCCO_2_, and the EV1000, were used simultaneously in a porcine model. In that study, the authors found a comparable bias and precision for CO (bias: 0.2 ± 0.3 L/minute, precision error 7%), GEDV (-11 L ± 80 ml, 14%), and EVLW (-5 ± 72 ml, 15%).

Volumetric preload indicators, such as GEDV, have been shown to be reliable indicators of cardiac preload [6,10,22-25] and have been successfully implemented into therapeutic strategies that may improve outcome [12]. EVLW, measured by single indicator dilution, is a reliable measure of pulmonary edema that has been validated against postmortem gravimetric measurement in animals [25], computer tomographic lung density measurements and double dye dilution [26], and, recently in a human autopsy study [27]. It is very sensitive even in small increases of pulmonary fluid content [9] and has been shown to be superior to other bedside measures of pulmonary edema [28]. Additionally, it is more specific, because only pulmonary edema and not pleural effusions are assessed. It can be used as an important diagnostic tool for detection and surveillance of acute lung injury [29] and as a prognostic parameter [10]. As with GEDV, EVLW has been successfully implemented into therapeutic algorithms [30].

A major limitation of the presented data is the lack of a third technique as a reference. In the present study, interchangeability of the two algorithms could be demonstrated within the limits that have been proposed for such analysis. Only comparisons with a true gold standard would allow individual testing of each algorithm. However, the use of a gold standard, for example, an epi-aortic flow probe [31], is not feasible at bedside in an ICU population. Moreover, an epi-aortic flow probe is only able to determine cardiac output; there is no gold standard for GEDV or EVLW. Another important limitation of the present study is that hemodynamic measurements were not performed simultaneously by the two devices with two thermistor-tipped femoral artery catheters. Consequently, it is not possible to compare all different system parts (for example, thermistor for blood and injectate temperature), as only VolumeView™/EV1000™ hardware was used to obtain raw hemodynamic data and the PiCCO_2_™ calculated CO, GEDV and EVLW from these data. The simulation approach was used to avoid an additional arterial puncture, indwelling catheter, and double measurements. Other researchers have employed comparable strategies, for example, for the comparison of pulse contour algorithms [31,32]. Our data are in line with that of an animal study using VolumeView™ and PiCCO™ catheters simultaneously [13], suggesting that no significant bias was introduced. However, one has to be careful with the interpretation, as the generally accepted threshold for interchangeability of a performance error below 30% [18] relies on the assumption of independent variances of the two methods. With the similar methods we used, this may not be the case. Additionally, the 30% threshold is based on the assumption that the precision of the reference technique is around 15%, while it may be lower for TPTD [33]. Consequently, if the percentage error for CO, GEDV, and EVLW would be close to 30%, the two measurements might actually not have been considered interchangeable. With a percentage error substantially below 30%, as demonstrated in this study, interchangeability can be safely assumed despite these methodological issues. Another limitation is that in a mixed population and without any predefined systematic intervention, GEDV, EVLW and CO are not evenly spread over the range of measurements, but are grouped around their median. For the trending analysis, this resulted in exclusion of roughly 50% of data points using the traditional concordance approach, even with a small exclusion zone of 10%. In contrast, the polar plot concept recently proposed by Critchley *et al. *[19,20] allowed the inclusion of all data points for trend analysis. In order to entirely avoid the application of an exclusion zone, the 30-degree radial limits were not used for interpretation of the results. Another minor limitation of the study is the high proportion of cardiac surgical patients (56%), which might limit the extension of the results beyond such a population. Finally, both systems display continuous cardiac output, derived from pulse contour analysis. These values are not part of this analysis, but will have to be considered when comparing the monitors in daily practice.

## Conclusions

In a mixed ICU population, and in a wide range of clinical situations, CO, GEDV and EVLW values assessed with the new VolumeView™/EV1000™ system are interchangeable with the current PiCCO™ method. The study was not designed to demonstrate any superiority of one algorithm over the other, but the higher precision of GEDV measurements when the VolumeView™/EV1000™ method was used suggests increased robustness of the algorithm against thermal noise and recirculation.

## Key messages

• TPTD is a less invasive technique for CO measurements; it does not depend on pulmonary arterial catheterization. In addition to CO measurements, TPTD additionally provides volumetric hemodynamic parameters (GEDV and EVLW)

• Recently, a new TPTD system has been developed that deploys a novel algorithm for GEDV and EVLW

• We compared measurements by that system to those by a PiCCO_2_™ in 72 critically ill patients

• The measurements by both systems agree closely, as well for absolute measures (bias, percentage error) as for measurements of change (concordance)

• For GEDV, the VolumeView Method had a higher precision

## Abbreviations

CE: coefficient of error; CO: cardiac output; Dst: downslope time; EVLW: extravascular lung water; GEDV: global end-diastolic volume; LOA: limits of agreement; m/f: male/female; Mtt: mean transit time; PAC: pulmonary artery catheter; RLOA: radial limits of agreement for trending; SD: standard deviation; TPTD: transpulmonary thermodilution.

## Competing interests

NK has received speaker's honoraria from Edwards Lifesciences and support for congress fees, CH has received speaker's honoraria and research grants form Edwards Lifesciences, Pulsion Medical Systems, and CSL Berhring, Berne, Switzerland. GM has received speaker's honoraria, consultant fees and a research grant from Edwards Lifesciences. AH has received speaker's honoraria from Edwards Liefesciences. KB has received consultant fees from Edwards Lifesciences. SR has received speaker's honoraria and consultant fees from Edwards Liefesciences. MG, RG, NS have no potential conflicts of interest to declare.

## Authors' contributions

CH, GM, AH, KB and SR conceived the study. NK, CH, RG, MS, NS and SR collected the data. NK and SR carried out the statistical analysis and drafted the manuscript. All authors read and approved the final manuscript.

## Supplementary Material

Additional file 1 **Details of conversion of EVLW formulas**. A step-by-step conversion to demonstrate strict mathematical conformity of the formulas used to calculate EVLW by the two devices (PiCCO2™ and Volume View™). Click here for file
