# Supplementary material for: Clinical validation of a new thermodilution system for the assessment of cardiac output and volumetric parameters
Source: Crit Care. 2012 May 30;16(3):R98. doi: 10.1186/cc11366 (PMC3580647; doi:10.1186/cc11366)

Additional file 1

This is the typical EVLW algorithm published for the PiCCO™ system (11).


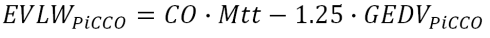
 (9)

Substitute GEDV with formula (2):


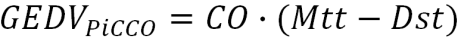
 (2)

and regroup:


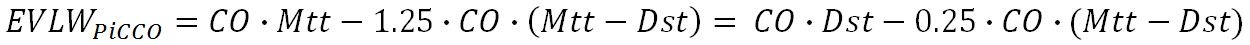
 (10)

According to formula (2):


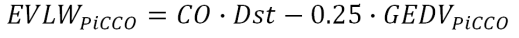


which is equivalent to the EVLW algorithm used by for the EV1000™ system:


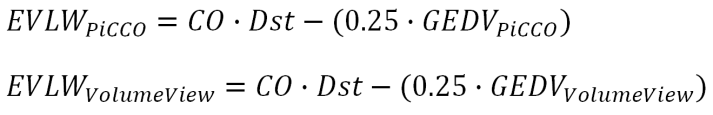

Supplement: Additional file 1 — Details of conversion of EVLW formulas. A step-by-step conversion to demonstrate strict mathematical conformity of the formulas used to calculate EVLW by the two devices (PiCCO2™ and Volume View™). [file cc11366-S1.DOCX]
